# Supplementary material for: Fast detection of slender bodies in high density microscopy data
Source: Commun Biol. 2023 Jul 19;6:754. doi: 10.1038/s42003-023-05098-1 (PMC10356847; doi:10.1038/s42003-023-05098-1)
Supplement: Supplementary file 2 — Supplementary Information [file 42003_2023_5098_MOESM2_ESM.pdf]

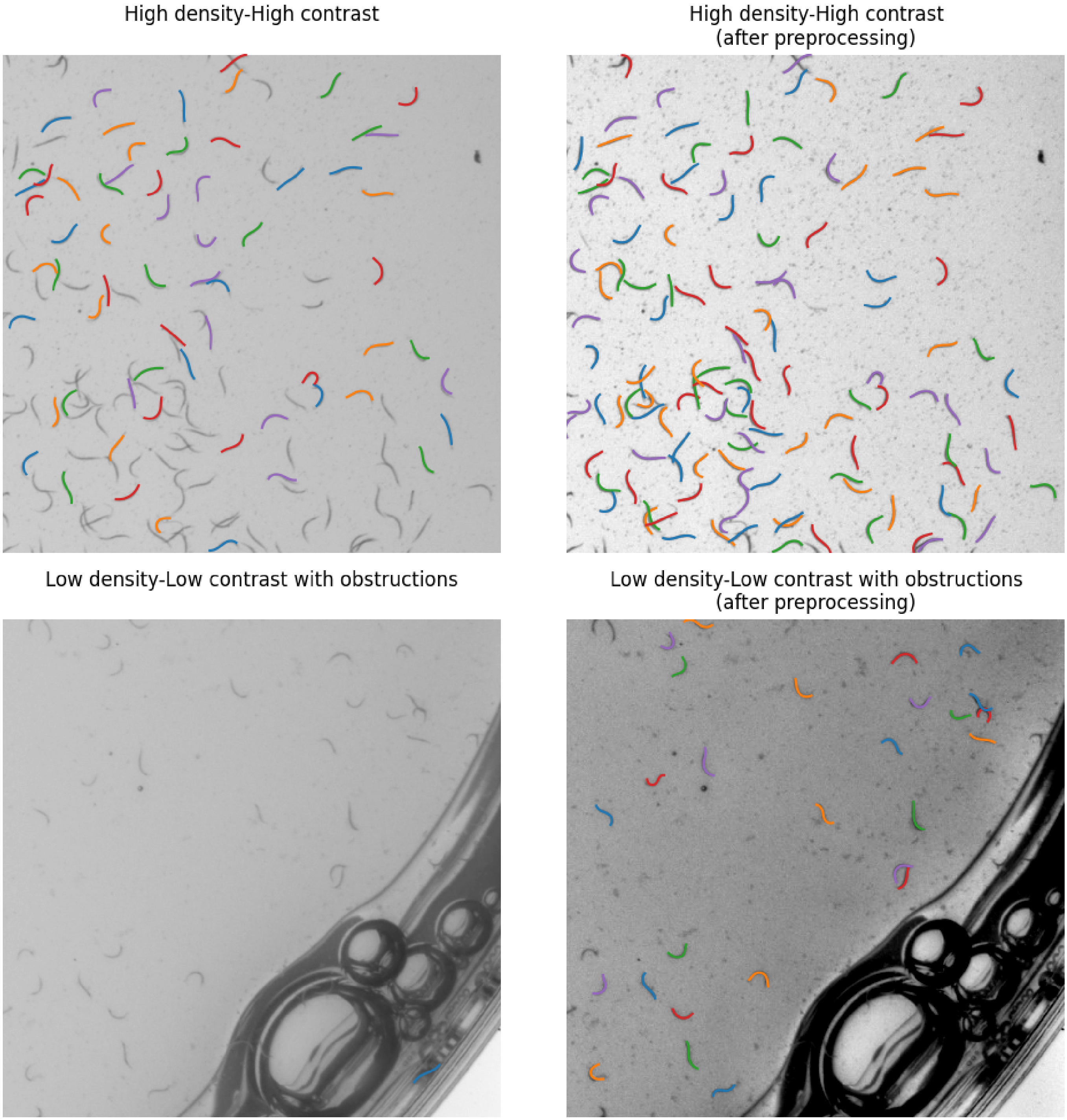

Supplementary Figure 1: Two examples of model output on raw data and on preprocessed data (CLAHE and intensity correction).
