# Supplementary material for: Fast detection of slender bodies in high density microscopy data
Source: Commun Biol. 2023 Jul 19;6:754. doi: 10.1038/s42003-023-05098-1 (PMC10356847; doi:10.1038/s42003-023-05098-1)
Supplement: Supplementary file 3 — Description of Additional Supplementary Files [file 42003_2023_5098_MOESM3_ESM.pdf]

Description of Additional Supplementary Files

File name: Supplementary Video 1
Description: Track of a 2736 x 2192 video

File name: Supplementary Video 2
Description: Track of a 512 x 512 region

File name: Supplementary Video 3
Description: Following a single worm in a track

File name: Supplementary Data 1
Description: The source data for graphs and charts
